# Research on Construction of High-Quality Application-Oriented Talent Cultivation System for Internet of Things Engineering: Based on Educational Psychology

**DOI:** 10.3389/fpsyg.2022.921840

**Published:** 2022-06-02

**Authors:** Yalin Nie, Haijun Wang, Baoluo Liu, Shangsen Yang

**Affiliations:** ^1^Department of Computer and Information Engineering, Luoyang Institute of Science and Technology, Luoyang, China; ^2^School of Mathematics and Statistics, Henan University of Science and Technology, Luoyang, China

**Keywords:** Internet of Things Engineering, high-quality, application-oriented, talent cultivation, educational psychology

## Abstract

High-quality talent cultivating Internet of Things (IoT) Engineering is the basis for the rapid development of IoT technology. To train high-quality application-oriented IoT technical talents, guided by educational psychology, this article conducts in-depth research; analyzes the characteristics of IoT Engineering; makes professional talent cultivating programs and cyclical adjustment plans; builds a high-quality teaching system based on the professional knowledge system of the IoT; explores the “spiritual level” and “psychological level” characteristics of teachers and students in teaching; highly integrates “Industry-University-Research-Competition” from the perspective of students, teachers, and colleges; infiltrates positive psychological cues appropriately; formulates the construction method of the “student teaching assistant” auxiliary system to enhance the efforts to promote learning by learning; and finally innovates the talent cultivating system for the IoT Engineering. The implementation results show that the students trained by this system have a solid foundation of knowledge related to IoT Engineering and strong engineering practice application, adaptability, and innovation ability.

## Introduction

Based on sensing technology, information recognition technology, communication technology, and control technology, the Internet of Things (IoT) realizes the connection among things and is one of the global strategic emerging industries. At the fifth session of the 11th National People’s Congress on 5 March 2010, the IoT was written into the Government Work Report for the first time. Since then, the industrialization process of IoT technology has been fully promoted, combining the development strategies of energy conservation, consumption reduction, green and low carbon with the sustainable development industry, food safety, disaster monitoring, modern logistics, intelligent transportation, and other industries as the entry point. The research and development of IoT technology promotes the development of devices, chips, materials, services, and other industries; forms an industrial cluster effect; promotes the integration of the undertaking of traditional industries and the independent innovation of emerging industries; and leads to the enduring demand for IoT technical personnel in the talent market. The talent cultivation of IoT has become the key to the sustainable and rapid development of the IoT.

To train technical talents for the IoT, universities in China have started to offer the major of IoT Engineering successively since 2010, and our university (Luoyang Institute of Science and Technology) also added the undergraduate major of IoT Engineering in 2014. Compared with the traditional established major, this major is an interdisciplinary subject, involving the integration of computer, automation, communication engineering, and other multidisciplinary knowledge. The development time is short, and the talent cultivation scheme is not mature. Therefore, the talent training system of IoT Engineering must be constantly improved and perfected based on the development needs of society and school (Chen et al., 2018; Gui et al., 2018).

Educational psychology (Slavin, 2016) mainly studies various psychological states, changes, and problems in the teaching process; helps educators find appropriate ways to deal with students’ psychological changes on the “spiritual level” and “psychological level”; further judges and analyzes students’ learning status and life ability; and optimizes students’ mental and psychological status by using positive psychological intervention and high-quality teaching design (Wang, 2021; Xiang, 2021). The introduction of educational psychology into all the aspects of teaching for IoT Engineering is of great help to train high-quality application-oriented talents.

## Development and Adjustment of Talent Cultivation Scheme

In view of the characteristics of interdisciplinary integration, the development of talent cultivation programs for IoT Engineering majors needs to fit social needs, school positioning, and teacher-student status analysis based on educational psychology. By investigating official websites of government departments, public institutions, large enterprises (Huawei, Alibaba, Tencent, etc.), and recruitment websites (such as landing.zhaopin.com and 51job.com), we can learn the types and levels of current demand for IoT talents, get the multidisciplinary demand for IoT technology, and determine the industry fields for talent cultivation. According to the college development plan, the key points of talent cultivation are determined: talent cultivation of IoT technology with big data in building materials industry. Questionnaires are used to obtain feedback on graduates’ employment, such as industry and salary. Shortcomings of existing talent cultivation programs are found while exploring the space for improving the quality of talent cultivation.

By investigating the IoT Engineering majors of other universities, such as Jiangnan University and Xidian University, and drawing on the talent cultivation experience of other universities, the cultivation program is set up mainly according to the combination of theoretical teaching and practical teaching, and the cultivation of students’ innovative consciousness and professional skills. At the same time, based on educational psychology, the current situation of teachers and students, the software and hardware environment, the existing scientific research, and competition basis of this major in our college are analyzed. Combined with the development plan of the college, the platform and direction are set up, and an application-oriented, student-oriented, and feature-oriented IoT technical talent cultivation system is built.

As the IoT technology is in the stage of rapid development currently, there are continuous changes in the knowledge system. Therefore, after the cultivation program is being formulated, it is necessary to repeatedly demonstrate and scrutinize the rationality of the theoretical and practical curriculum system of the IoT Engineering, and the teaching plan of each course should be formulated and improved for the integration of courses.

Therefore, after the cultivation plan is being formulated, it is necessary to repeatedly demonstrate and deliberate the rationality of the theoretical and practical curriculum system of the IoT Engineering, and develop and improve the teaching plan of each course for curriculum integration. In addition, guided by Engineering Education Professional Accreditation Standards (Liu et al., 2020), based on educational psychology constructivism, we keep in step with the frontiers of IoT technology, follow the “four years a major change, two years a minor change, slightly change every year” cultivation scheme maintenance process, adjust the curriculum system, and reform the practical training timely, which make the teaching of IoT technology as high quality and ensure the cultivation of the students as efficient and high quality.

**FIGURE 1:**
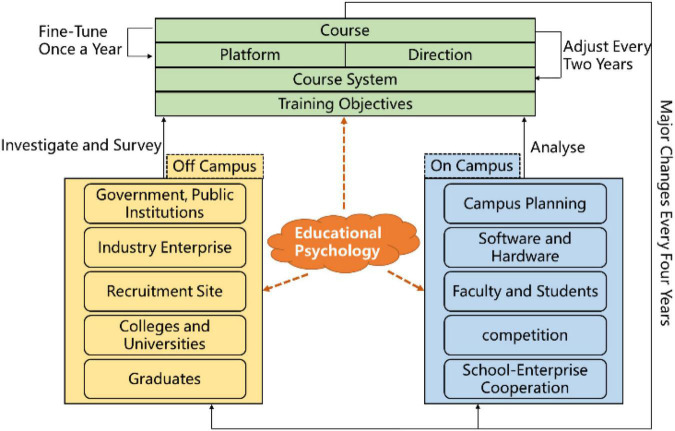
Development and adjustment process of talent cultivation program.

Figure 1 shows the development and adjustment process of the talent cultivation program of our school based on educational psychology. This process aims to make the training program prepared and adjusted to guide various teaching activities effectively. The trained students could master the knowledge and skills in the professional field of IoT, and they can analyze, research and design solutions to solve the problems of the IoT in building materials big data research and development. It helps students to have unique high-quality thinking mode, strong engineering practice ability and lifelong learning ability with respect to the IoT Engineering major, leading to strong core competitiveness in the workplace.

## Construction of High-Quality Teaching System

### Professional Knowledge System of Internet of Things Engineering

**FIGURE 2:**
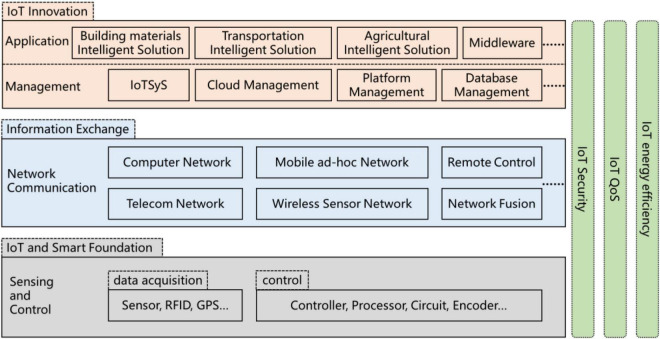
Internet of Things Engineering professional knowledge system.

Constructivism in educational psychology believes that the essence of learning is a process in which students construct their own framework by using the knowledge they have learned (Liu G., 2017; Liu, 2021). Therefore, it is critical to determine the knowledge system of IoT Engineering. IoT is a technology that implements the connection between things and things. Specifically, it refers to using information sensing equipment and control equipment to connect objects to networks according to agreed protocols to exchange information among things, and finally realizing intelligent identification, positioning, tracking, supervision, etc., involving the equipmentization of common objects, the interconnection of autonomous terminals, and the intelligence of pervasive services. From the perspective of layering, the IoT knowledge system from bottom to top successively is sensing and control layer, network communication layer, and application service layer, where the application service layer can be subdivided into a management sublayer and an application sublayer (as shown in Figure 2). Sensing and control layer involves various types of sensors (temperature and humidity, pressure, direction, liquid level, infrared, etc.), processors, controllers, circuits, data coding, identification technology, etc., which are the basis for the object being equipment and intelligence. Network communication layer involves all kinds of network technologies, such as computer network, telecommunication network, mobile *ad hoc* network, and wireless sensor network, which is the core of object–object communication. The application service layer involves various implementation technologies for electronic and intelligent services. The management sublayer is associated with technical knowledge such as IoT systems, platform management, cloud management, and database management. The application sublayer is associated with various intelligent solutions and middleware, industry-specific technologies, etc., which is the key to implement the innovative application of IoT technology. All IoT technical knowledge layers relate to IoT security, QoS, and energy efficiency management.

### Cultivation Goals

Our college is one of the second batch of demonstrative application-oriented undergraduate colleges in Henan Province and has clearly positioned itself as “dual service”: serving the development strategy of the national building materials industry and serving the economic and social development of Luoyang. The development of talent cultivation goals is related to the characteristics and service orientation of our college closely and focuses on the cultivation of innovation and application capabilities. At present, combined with educational psychology constructivism, the general cultivation goal of our IoT Engineering major is set as follows.

The IoT major aims to gain high-quality application-oriented talents. These talents meet the needs of the modern information technology industry; serve the regional economic and social development of Luoyang; develop in an all-round way in morality, intelligence, physique, beauty and labor, master the basic theory, knowledge, and skills of IoT Engineering; have good technical innovation and engineering practice ability; and are able to engage in application development, engineering design, system management, and operation maintenance in the fields related to IoT technology in building materials industry.

After graduation, students of this major are expected to achieve the following specific goals through 5 years of practice:

(1) Meet the needs of the development of innovative talents, with all-round development of both political integrity and ability, showing good humanistic quality, professional ethics, and social responsibility.
(2) Have a solid mathematical foundation and a good basis of natural science.
(3) Master the professional knowledge and skills of the IoT; be able to analyze, research, and design solutions; solve the application problems of the IoT in the research and development of building materials with big data; have strong engineering practical ability and unique thinking mode of the IoT Engineering; and become the technology backbone.
(4) Have engineering organization ability, teamwork ability, and international vision.
(5) Have lifelong learning ability and be able to update and expand their knowledge and ability through appropriate ways.

### Curriculum System

Curriculum system is the core of higher education, reflecting social needs, scientific knowledge, and ability development (Jiang et al., 2021). The construction of the curriculum system of IoT Engineering is based on the knowledge system of IoT technology and professional cultivation goals. The setting of professional courses inevitably involves three aspects, namely, sensing and control, network communication, and IoT application services, and integrates the industry characteristics and the social needs for students’ abilities.

Constructivism holds that “learners do not transfer knowledge from the outside world to memory, but construct new understandings based on existing experience through interaction with the outside world.” To cultivate high-quality application-oriented talents, the curriculum system must be able to cultivate students with correct world outlook, outlook on life, and values.

**FIGURE 3:**
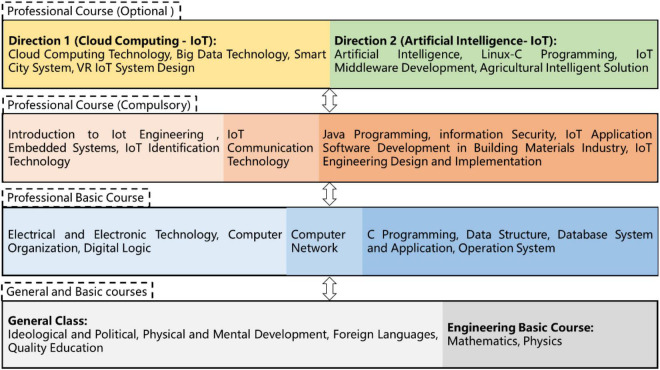
Curriculum system of IoT Engineering.

To cultivate students majoring in IoT Engineering to have a good sense of appreciation, be rich in knowledge and experience and get excellent ability, at present, the curriculum system of this major should include four categories, namely, general courses, basic engineering courses, professional basic courses, and professional courses, as shown in Figure 3.

General courses mainly include Ideological and Political Theory Courses, Physical and Mental Development Courses, Language and Tool Courses, and Quality-Oriented Education Elective Courses. At present, the Ideological and Political Theory Courses in our college include Morality and Law, Basic Principles of Marxism, Introduction to Mao Zedong Thought and the Socialist Theory with Chinese Characteristics, and Outline of Contemporary and Modern Chinese History. Physical and Mental Development Courses include Physical Education, Military Theories, Mental Health Education for Undergraduate, Career-Ready Skills with Innovation, and Entrepreneurship Practice. Language and Tool Courses include College English and College Applied Writing. Quality-Oriented Education Elective Courses include courses about humanities and art, social science, natural science and technology, economic management, and general development of foreign languages (Oral English and Japanese, translation and speech).

Basic engineering courses for IoT major mainly include mathematics and physics. Mathematics courses include Advanced Mathematics I, Linear Algebra I, Probability and Statistics I, and Discrete Mathematics. Physics courses include College Physics II and College Physics Experiment.

Professional basic courses mainly focus on the basic theory and program design of IoT technology, aiming to train the student to gain IoT technology learning foundation and prepare for the study of knowledge required by the design and development of IoT systems. This type of course covers Electrical and Electronic technology, Program Design of C Language, Computer Organization and Architecture, Data Structures, Database Systems and Applications, Computer Networks, Operating Systems, and Digital Logic.

Professional courses are divided into professional required courses and professional elective courses, which mainly train students to have the ability of IoT system design, development, management, and maintenance; to serve the IoT application in building materials industry; and to provide direction for employment and further study. Professional required courses for IoT mainly include Specialty Introduction for IoT Engineering, Java Programming, Information Security, IoT Communication Technology, Embedded System, IoT Identification Technology (college-enterprise), IoT Application Software Development for Building Materials Industry, and IoT Engineering Design and Implementation. The professional elective courses are divided into two directions: Direction One (Cloud Computing-IoT): Cloud Computing Technology (college-enterprise), Big Data Technology, Smart City System, and IoT VR Design; Direction Two (Artificial Intelligent-IoT): Artificial Intelligence, Linux-C Programming, IoT Middleware Development, and Smart Agriculture System.

At present, the curriculum system of IoT Engineering major in our college highlights the knowledge that students of this major should master in quality-oriented education and engineering education. Professional basic courses constitute the most core basic knowledge of this major, and the setting of professional courses reflects the common requirements of IoT technology application ability (sensing/control, communication, and application) and the training focus of IoT Engineering major in our college.

### Practice Teaching

Our college is positioned as application-oriented, and its teaching activities focus on “application,” aiming at cultivating high-quality application-oriented talents with strong social adaptability and competitiveness. IoT Engineering itself is closely related to practical application, so practice teaching is the key to make students digest kinds of theoretical knowledge (Wang et al., 2019; Chen et al., 2021) and apply them to practical application and is the top priority in the training process of application-oriented talents, and the quality of its implementation directly affects the training quality of application-oriented talents.

**FIGURE 4:**
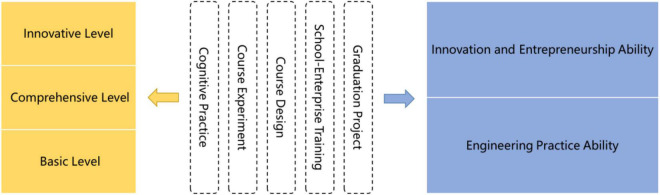
“Three-level and Five-category” practice teaching system.

The application ability of students majoring in IoT Engineering includes engineering practice ability and innovation and entrepreneurship ability (Cui et al., 2019). Around cultivating the two kinds of abilities, we build a “Three-level and Five-category” practice teaching system. Among them, “Three-level” refers to basic, comprehensive, and innovative practice teaching, and “Five-category” refers to five categories of practice teaching activities, including professional cognitive practice, course experiment, course design, college-enterprise practice training, and graduation project, as shown in Figure 4.

The IoT Comprehensive Experiment Center of our college is one of the Experimental Teaching Demonstration Centers in Henan Province. It has an IoT experience experiment platform, a communication experiment system, a smart experiment platform of agricultural machinery, Cisco network routers and switches, high-performance servers and computers, optical fiber fusion splicers, IoT Comprehensive testers, FLUKE network testers, and embedded experiment boxes and provides experiment environment for professional experiments such as a single-chip microcomputer, embedded system, RFID, wireless sensor networks, communication and switch, upper computer, cloud, and AI. We utilize educational psychology to set up three-level experiment courses. These courses incorporate the application of the IoT in the building materials industry, smart agriculture, and advanced manufacturing industry. Step by step, they can mobilize the initiative of students in the experiment/experimental training and can cultivate engineering practice ability in cement production simulation, smart livestock farm, industrial Internet, and other industries, highlighting the characteristics of the IoT Engineering major of our college and the good teaching quality.

Experiment is the foundation for cultivating ability to apply knowledge flexibly, and practical training is the basis for students to improve their personal abilities and possess engineering practice and innovation/entrepreneurship capabilities. By rationally setting up on-campus practical training courses, using educational psychology scientifically to analyze students’ practical training status and arrange corresponding practical training details, students can consolidate their learned knowledge and cultivate their practical ability in the actual trial production environment. Based on educational psychology, our college guides students to contact, understand, and serve the society through reasonable planning of corporate training, IoT project engineering design, and graduation project; and cultivate students’ IoT comprehensive practical ability, especially the service and innovation capabilities of IoT in building materials industry with big data. Our college has set up a practical training center for IoT and further built practical training and scientific research platforms such as “Cisco Network Technology Institute,” “Huawei Institute of Information and Network Technology,” “Renesas Electronic Joint Laboratory,” and “Shuguang Ruiyi Big Data Innovation Center.” At the same time, our college has signed eight off-campus practice bases with Shenzhen Xunfang Communication Technology Co., LTD., Wuxi NIIT (China) Institute of Service Outsourcing, and other related enterprises, to ensure the quality of on-/off-campus practical training courses in terms of software, hardware, and teachers.

## Deep Integration of “Industry-College-Research-Competition”

“Industry-College-Research-Competition” (ICRC) refers to the cooperation between colleges and enterprises (Xiong, 2021), which integrates and gives full play to the advantages of teaching, scientific research, competition, and enterprise resources to carry out teaching activities, improving teaching quality, and cultivating application-oriented talents with strong theoretical and practical abilities (Liu C. B., 2017; Zhang, 2020).

**FIGURE 5:**
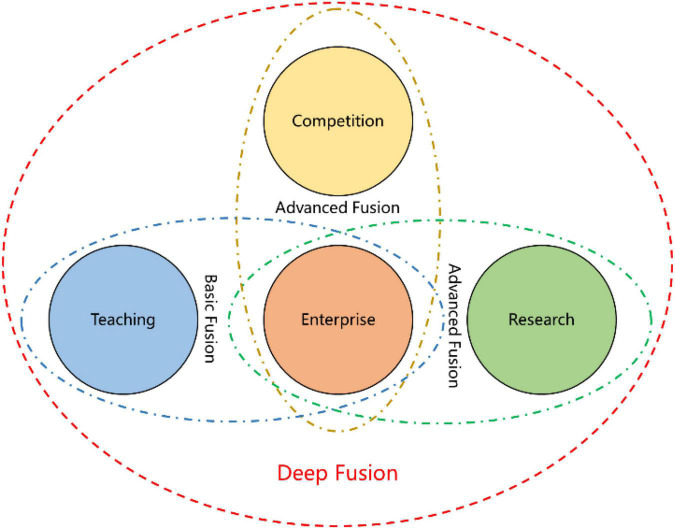
Deep integration of ICRC.

To achieve the deep integration of ICRC (as shown in Figure 5), we organized teaching activities according to the “spiritual level” and “psychological level” characteristics of teachers and students. (a) Deepen the cooperation with enterprises related to IoT technology, implement the dual integration of resources from college and enterprises off and on line, and efficiently combine theory teaching and experimental teaching, on-campus practice teaching and social practice teaching. (b) Encourage IoT Engineering teachers to carry out temporary employment in enterprises, to cooperate with enterprises to carry out scientific research or directly participate in enterprise projects, to guide students participating in scientific research and enterprise activities, to expand students’ horizons, to stimulate students’ interests on scientific research, and to cultivate students’ abilities in solving practical engineering problem, and, at the same time, to improve teachers’ own IoT Engineering literacy. (c) Take participation in competitions as a compulsory subject for students, and teachers and corporate mentors guide students jointly to participate in various IoT-related competitions, while through competitions inspiring the report, construction, and expansion of IoT practice projects, cultivating students’ awareness of competition to enhance employment competitiveness, and enhancing professional comprehensive strength and the ability of IoT Engineering to serve the local economy and building materials industry in Luoyang.

Teachers guide students to construct their frameworks in ICRC. At present, the deep integration of ICRC of IoT Engineering major is shown as follows:

(1) *Student perspective*: In four years of college, complete a scientific and technological research in the field of IoT independently, complete several experimental projects related to the needs of enterprises and competition, participate in scientific research activity for teachers at least 1 time, participate in enterprise IoT projects at least 2 times, complete a comprehensive innovative experimental project cooperatively, and participate in IoT-related competition at least 1 time.
(2) *Teacher perspective*: Link their own scientific research with enterprises (Yang et al., 2021), find the breakthrough point of scientific research from enterprise engineering projects with scientific research results serving actual engineering research, guide students to participate in scientific research activities, such as composing engineering patents and papers and coding engineering projects, design experiments, internships, and practical training around IoT Engineering practical applications, often invite enterprise engineers to participate in the design and implementation of teaching activities, guide students to participate in various IoT-related competitions, organize competition training for students, follow-up the IoT Frontiers, explore the application points of IoT technology related to building materials with big data and integrate them into the usual teaching, and always reflect the power of connecting theory with practice.
(3) *College perspective*: Establish good and stable college-enterprise cooperation with IoT-related enterprises, take the market demand as the guidance, serve Luoyang economy and building materials industry, explore the cultivation mode based on the ICRC integration for the training of high-quality applied talents, improve multi-evaluation subsystem reflecting the characteristics of ICRC with respect to the curriculum system, teaching team, college-enterprise joint practice bases and competition training mode, and construct the talent cultivation subsystem of ICRC integration, carrying out efficient and high-quality teaching work.

## Construction of “Student Teaching Assistant” Auxiliary Mechanism

The relevant theories of educational psychology point out that the teacher–student relationship is the most basic and important interpersonal relationship when implementing teaching activities, and the teacher’s attitude toward students affects the quality of teaching directly. Therefore, the cultivation of high-quality application-oriented talents is dependent on the harmonious teacher–student relationship and the smooth development of student-centered teaching activities (Ji et al., 2019). At present, the IoT Engineering major of our college generally conducts teaching activities in co-classes. The minimum co-class (two co-class) is at least 55 students, and the number of students of a large co-class can be hundreds. Due to the large number of students in co-class, the following causes occur: (1) It is difficult for teachers to give lectures to students of all levels, and the interaction between teachers and students in class lacks pertinence. (2) The management of theoretical teaching is difficult, and there are many mobile phone phubbing students, so it is difficult to mobilize the class atmosphere. (3) There are many problems in practical teaching. For the large number of students who need experiment/practice/training guidance, meticulous live guidance cannot be given to each student. The more detailed experiment/practice/training guidebooks encourage students to think inertly, and there are many “goofing-off” practices, which affect the cultivation of practical ability seriously.

By studying the cooperative learning theory in educational psychology, our college has set up a mechanism “Student Teaching Assistant (STA)” (Nan et al., 2016; Zhang and Qin, 2019; Zhang and Dai, 2020). This mechanism selects students from the IoT Engineering majors to assist in the implementation of various teaching activities, explores the potential of students, and helps learning with students, which is one of the effective means for the college to build a harmonious teacher–student relationship. There are three categories in STA, namely, course assistant (CA), practice assistant (PA), and competition and research assistant (CRA). STA mechanism does not simply divide students into teaching assistants and ordinary students. The simple hierarchical management for students will only weaken the teacher–student relationship and strengthen the blocking of teacher–student communication in teaching activities. Generalized STA makes students complete a kind of teaching assistant task at least once in the 4-year study and strengthen the communication, cooperation, and collision between teachers and students, while teachers implement positive energy psychological hint reasonably and finally establish a healthy development for the relationship between teaching and learning. The teaching assistant activities promote students’ ability of hands-on practice, organization, cooperation, and innovation and cultivate engineering application-oriented talents with solid theoretical foundation.

Course assistants are responsible for maintaining classroom discipline; supervising pre-class preview, after-class review, and homework; and organizing flipped classrooms. CAs’ solid efforts in learning are conducive to creating a good atmosphere for class learning. They assist teachers to detect students’ psychological changes timely; promote healthy competition in learning in the class; encourage all students to develop good habits for listening to classes, previewing and reviewing; improve the utility of flipped classroom teaching activities such as teaching video watching and cooperative discussion; organize curriculum knowledge of IoT Engineering in series by mind mapping and other methods; and organize course knowledge competition. CAs give full play to the role of mentoring and guiding poor students and make progress together. They are selected within the same grade. Each course has 3–4 CAs, and each CA is responsible for a study group of less than 10 students. With the help of CAs, the phenomenon of “phubber” in class, homework copying, etc., can be reduced. Good college study habits can be established, and students are more active and solid in learning the theoretical knowledge related to IoT, which is many, complex, and difficult.

In addition to experimental classes, our college has an open system to facilitate students to use the laboratory to complete practical tasks after class. One role of PA is the laboratory-assisted management. PAs assist teachers to urge students to maintain a good experimental environment and follow laboratory discipline consciously, and this mechanism cultivates students’ sense of ownership about the laboratory. Another role of PA is to assist teachers to guide various IoT-related experiments, internships, and practical training courses. PAs take the initiative to solve common problems in practice for other students, promote mutual assistance among students, discover and summarize problems, and give feedback to teachers to solve them in a timely manner. PAs are produced from the students in senior grades and the same grade, among which the PAs from senior grades assist in the practice and training and the PAs from the same grades assist in course experiments. With the help of PAs, the interaction between teachers and students is enhanced, so that detailed practical guidance can cover each student, which is conducive to the cultivation of students’ practical ability.

Competition and research assistants are mainly responsible for the collection and promotion of competition information, the auxiliary organization of competition training, the leading participation of enterprise projects, and teachers’ scientific research projects (such as IoT software coding and project data collation). They assist teachers to optimize the allocation of personnel and resources for competition and scientific research and shorten the distance between competitions/projects and students. Besides, they take themselves as examples to drive all the students majoring in IoT to learn the frontier knowledge. The selection of such assistants is based on the awards of the competition, and their responsibilities are determined according to the award level. Students with low levels of awards provide introductory training to lower grade students (such as IoT competition classification, participation process, and competition video production). High-level award-winning students assist teachers in training and guiding IoT technology competitions (such as embedded development, cloud server configuration, transmission protocol implementation, and upper computer coding). In addition, all CRAs participate in the project as team leaders responsible for the implementation of sub-tasks and the management of student members. With the help of CRAs, students are encouraged to form a healthy and upward competitive psychology and stimulate their innovative consciousness. In this way, competitions and participation in scientific research activities can become the norm for the IoT Engineering major, and students can develop their ability to discover, analyze, and solve engineering application problems.

## Teaching Results

In 2014, our college started to recruit students for the IoT Engineering and followed the above methods to build a talent cultivation system. At present, certain results have been achieved in cultivating high-quality applied talents.

**FIGURE 6:**
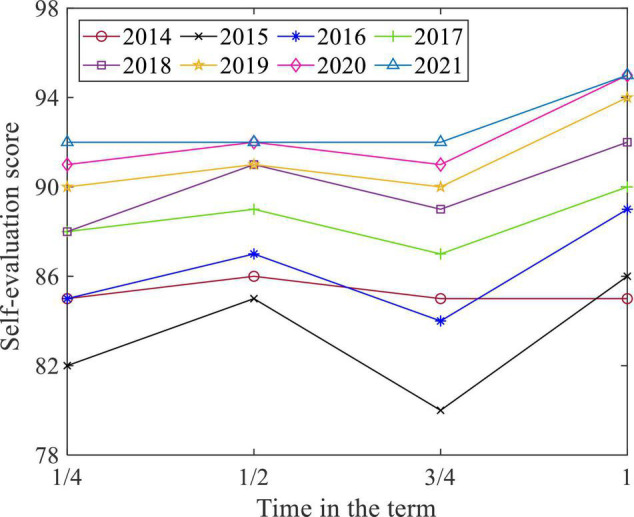
Student self-evaluation in learning.

To check students’ subjective initiative, questionnaires for students’ self-evaluation in learning are released at the 1/4 semester, 1/2 semester, 3/4 semester, and the end of the semester. (The full score of this type of questionnaire is 100. The higher the score is, the better the students’ subjective initiative in learning is.) The scores of the questionnaires from 2014 to 2021 were sorted out, and the results are shown in Figure 6. The students’ subjective initiative in learning was poor in 2014, but it improved year by year and was almost the same in 2019–2021, which shows that the implementation of various talent cultivation methods can effectively guide students in learning actively.

**FIGURE 7:**
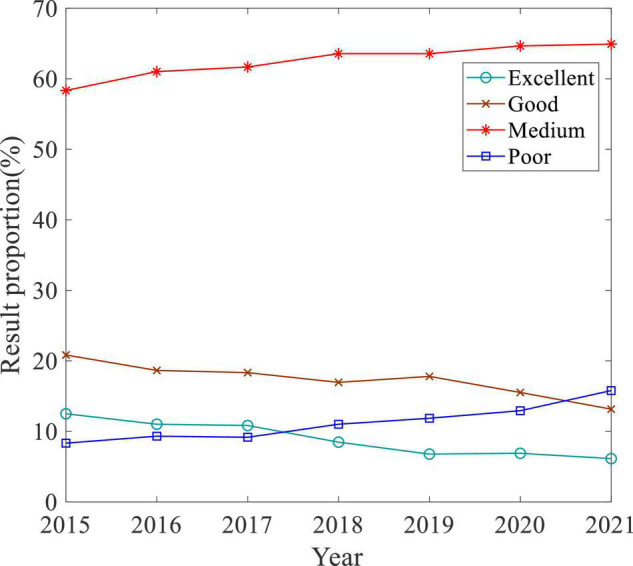
Evaluation of experiment and practice courses.

The execution quality of experiment and practice directly affects the quality of IoT Engineering talent cultivation. Collecting the assessment results of experiments and practice sessions, the average proportions of excellent, good, medium, and poor grades are calculated separately according to Eq. 1 every year from 2015 to 2021, and Figure 7 is obtained:

$$r_{L}=\frac{\sum_{i=1}^{m}E\,x\,p\,L_{i}+\sum_{j=1}^{n}\text{Pra}\,L_{j}}{\sum_{i=1}^{m}C_{i}+\sum_{j=1}^{n}P_{j}},$$

(1)    L={Excellend,Good,Medium,Poor},

where *m* and *n* are the number of courses and practice sessions, respectively; Exp *L*_*i*_ and Pra *L*_*j*_ are the number of students who gain grade *L* in experiments attached to course *i* and practice session *j* separately; and *C*_*i*_ and *P*_*j*_ are the number of students attending the course *i* and practice session *j*, respectively.

Figure 7 shows that the proportion of students who did not perform well in experiment and practice (poor, intermediate level) decreases with the improvement of the talent cultivation system, and most students were able to complete experiments and practical tasks well.

**FIGURE 8:**
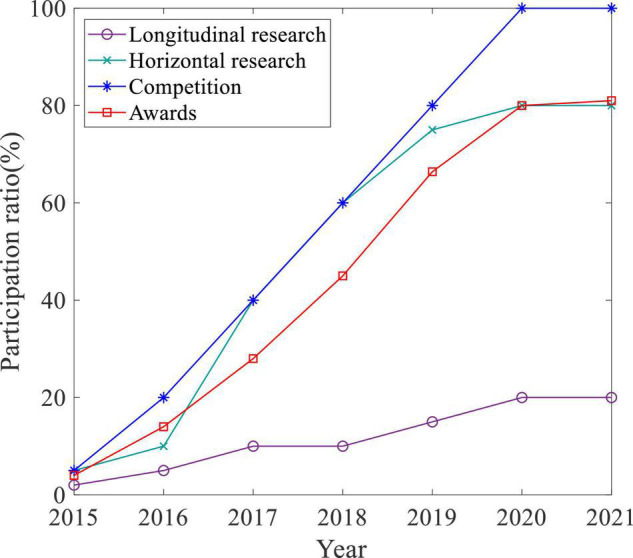
Competition and scientific research participation.

Participating in competitions and teachers’ scientific research is an effective way to enhance the core competitiveness of students. We counted the number of students who take competition and scientific research in IoT Engineering, with results shown in Figure 8. From 2015 to 2021, the ratio of participants and winners in various competitions has increased year by year. By 2021, all students participated in teachers’ scientific research more or less according to their ability, among which there are more horizontal projects and less vertical scientific research. Competition and scientific research promote more exchanges between teachers and students, making the relationship more harmonious and stimulating students’ competitiveness.

## Conclusion

Students are the objects of talent cultivation and the core of teaching activities, and their psychology directly affects the training quality. To this end, we can use educational psychology with student learning psychology as the core to improve the cultivation quality of application-oriented talents in IoT Engineering.

The construction of a high-quality application-oriented talent cultivation system for IoT Engineering involves many aspects. We analyze the existing knowledge architecture of IoT technology according to educational psychology. Based on the characteristics of the IoT major, we provide the formulation and adjustment strategies of talent cultivation programs and then put forward the idea of building a high-quality talent cultivation teaching system based on constructivism. Considering the psychological characteristics of teachers and students, we explore the deep integration mode of industry, college, research, and competition. Finally, we construct an STA auxiliary mechanism using a positive energy psychological hint. To sum up, all of these conduct the cultivation of high-quality application-oriented talents for IoT Engineering.

The rapid and sustainable development of IoT technology determines that it is a long way to cultivate high-quality application-oriented talents for IoT Engineering. In the future, efforts should be made to integrate educational psychology and teaching practice to obtain more efficient and high-quality methods of training application-oriented talents.

## Data Availability Statement

The original contributions presented in the study are included in the article/supplementary material, further inquiries can be directed to the corresponding author.

## Author Contributions

YN: investigation, writing original draft, data analysis, and method design. HW: applied research in educational psychology, investigation, and method design. BL: editing, writing, checking, and data collection. SY: implementation plan of educational psychology. All authors contributed to the article and approved the submitted version.

## Conflict of Interest

The authors declare that the research was conducted in the absence of any commercial or financial relationships that could be construed as a potential conflict of interest.

## Publisher’s Note

All claims expressed in this article are solely those of the authors and do not necessarily represent those of their affiliated organizations, or those of the publisher, the editors and the reviewers. Any product that may be evaluated in this article, or claim that may be made by its manufacturer, is not guaranteed or endorsed by the publisher.
